# Dataset of banana leaves and stem images for object detection, classification and segmentation: A case of Tanzania

**DOI:** 10.1016/j.dib.2023.109322

**Published:** 2023-06-16

**Authors:** Neema Mduma, Judith Leo

**Affiliations:** Department of Information and Communication Sciences and Engineering, P o Box 447, Tengeru, Arusha Tanzania

**Keywords:** Banana, Black Sigatoka, Fusarium wilt race 1, Leaves, Stem, Image

## Abstract

Banana is among major crops cultivated by most smallholder farmers in Tanzania and other parts of Africa. This crop is very important in the household economy as well as food security since it serves as both food and cash crops. Despite these benefits, the majority of smallholder farmers are experiencing low yields which are attributed to diseases. The most problematic diseases are Black Sigatoka and Fusarium Wilt Race 1. Black Sigatoka is a disease that produces spots on the leaves of bananas and is caused by an air-borne fungus called *Pseudocercospora fijiensis*, formerly known as *Mycosphaerella fijiensis*. Fusarium Wilt Race 1 disease is one of the most destructive banana diseases that is caused by a soil-borne fungus called *Fusarium oxysporum* f.sp. Cubense (Foc). The dataset of curated banana crop image is presented in this article. Images of both healthy and diseased banana leaves and stems were taken in Tanzania and are included in the dataset. Smartphone cameras were used to take pictures of the banana leaves and stems. The dataset is the largest publicly accessible dataset for banana leaves and stems and includes 16,092 images. The dataset is significant and can be used to develop machine learning models for early detection of diseases affecting bananas. This dataset can be used for a number of computer vision applications, including object detection, classification, and image segmentation. The motivation for generating this dataset is to contribute to developing machine learning tools and spur innovations that will help to address the issue of crop diseases and help to eradicate the problem of food security in Africa.

**Specifications Table**

**Table:** 

Subject	Applied Machine Learning
Specific subject area	Computer vision techniques for the detection of crop diseases affecting banana specifically Black Sigatoka and Fusarium Wilt Race 1
Type of data	Image
How the data were acquired	13-megapixel smartphone cameras from Samsung Galaxy 01 were used to collect the data. On the smartphones, the Open Data Kit (ODK) software AdSurv was installed in order to take pictures of the banana leaves and stems in the field. Banana image data were categorized as either healthy or afflicted by Fusarium Wilt Race 1 or Black Sigatoka. The farmers and researchers participated in the data collection process while plant pathologists and agricultural extension officers were responsible for the quality check.
Data format	Raw
Description of data collection	Over the range of six months from February 2021 to July 2021, images were collected in the field. The two detected diseases that are mostly affecting productivity were to be taken into consideration when compiling the dataset of banana disease diagnostics. By examining the caption for the banana image sample, the names of each disease in the dataset were identified.
Data source location	• Institution: The Nelson Mandela African Institution of Science and Technology (NM-AIST), The International Institute of Tropical Agriculture (IITA) • City/Town/Region: Arusha • Country: Tanzania
Data accessibility	Repository name: Harvard Dataverse Data identification number: doi:10.7910/DVN/LQUWXW Direct URL to data: https://dataverse.harvard.edu/dataset.xhtml?persistentId=doi:10.7910/DVN/LQUWXW

## Value of the Data

• Machine learning models for early detection of Black Sigatoka and Fusarium Wilt Race 1 diseases that affect productivity can be trained using this dataset.
• Researchers in the field of machine learning can use the collected imagery dataset of bananas to develop the end to end technological solutions to issues facing the agricultural sector.
• A variety of computer vision tasks, including object detection, classification and image segmentation can be facilitated by the generated dataset.
• To the best of our knowledge, this is among the biggest publicly available dataset on bananas in Tanzania, and the dataset comprises every potential case.

## 1 Objective

The aim of generating a banana dataset is to establish the foundation for African data repositories that will facilitate research activities for the artificial intelligence and machine learning researchers in the continent. The generated imagery dataset will provide an end to end machine learning solutions that will help to address the issue of food security and eradicating hunger in Tanzania and other parts of Africa. A variety of machine learning use cases such as classification and object detection can be delivered by the annotated image samples generated from this dataset. The dataset can be used by other researchers to simulate the spread of banana diseases, which would ultimately aid in the creation of resistant crop varieties required to solve the Sub-Saharan African continent's food security issue.

## 2 Data Description

**Fig. 1:**
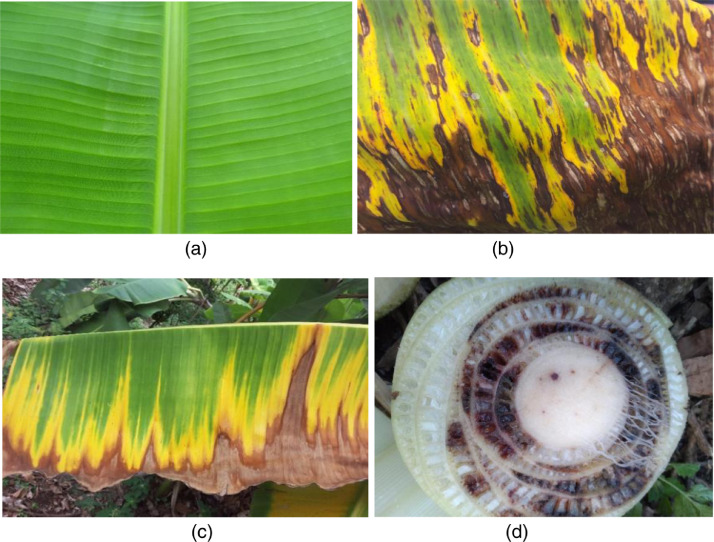
Image sample of banana (a) healthy (b) Black Sigatoka (c) Fusarium Wilt Race 1- Leaf (d) Fusarium Wilt Race 1- Stem.

An imagery dataset of healthy banana crop as well as those affected by Black Sigatoka and Fusarium Wilt Race 1 gathered from Tanzanian farms is presented in this article. The dataset has a total of 16,092 labeled images with 1024 × 768 pixels in jpeg format with a label indicating the name of the image based on the image number. In the repository, data were uploaded into 6 separate folders; 2 folders of healthy data submitted in zip format, 2 folders of Black Sigatoka data submitted in zip format, and 2 folders of Fusarium Wilt Race 1 data submitted in zip format. Also, all folders were named to indicate their corresponding image class. HEALTHY folders contain all images of healthy banana leaves, BLACK SIGATOKA_1 and BLACK SIGATOKA_2 folders contain images of banana leaves and stems affected by Black Sigatoka and FUSARIUM WILT_1 and FUSARIUM WILT_2 folders contain images of banana leaves and stems affected by Fusarium Wilt Race 1. Images were separated into different folders to allow easy uploading and downloading of data. Fig. 1 shows the sample of healthy banana, and those affected by Black Sigatoka and Fusarium Wilt Race 1.

## 3 Experimental Design, Materials and Methods

### 3.1 Field data collection

Imagery data of banana leaves and stems were collected by the Nelson Mandela African Institution of Science and Technology (NM-AIST) and the International Institute of Tropical Agriculture (IITA) located in Arusha, Northern Tanzania. Open Data Kit (ODK) application called AdSurv installed on Samsung Galaxy 01 smartphones with 13-megapixel were used to capture images of banana leaves and stems. Random sampling method was used to identify the plots for data collection and images for dataset generation. Farmers and researchers participated in the data collection process, while the issue of quality check was done by plant pathologists and agricultural extension officers. Banana imagery data were collected in six months from February 2021 to July 2021, and involved farms in Kagera, Arusha, Dar es Salaam, Kilimanjaro and Mbeya regions. The five regions were specifically chosen by taking into account the banana production and diseases prevalence [1].

### 3.2 Data preprocessing

The collected data were cleaned, renamed and annotated before uploading them to the open-access repository. Using VisiPics software, duplicate images discovered during data cleaning were eliminated [2]. If there are any duplicates left, the number should be so low as to not significantly affect training or testing [3]. The dataset could include unique images that aren't considered duplicates yet that are remarkably similar. Table 1 presents a number of banana images before and after removing duplicates.

**Table 1:** Banana dataset before and after removing duplicates.

Class name	Before removing duplicates	After removing duplicates
Healthy	6624	5628
Black Sigatoka	6899	5767
Fusarium Wilt Race 1	5877	4697

**Fig. 2:**
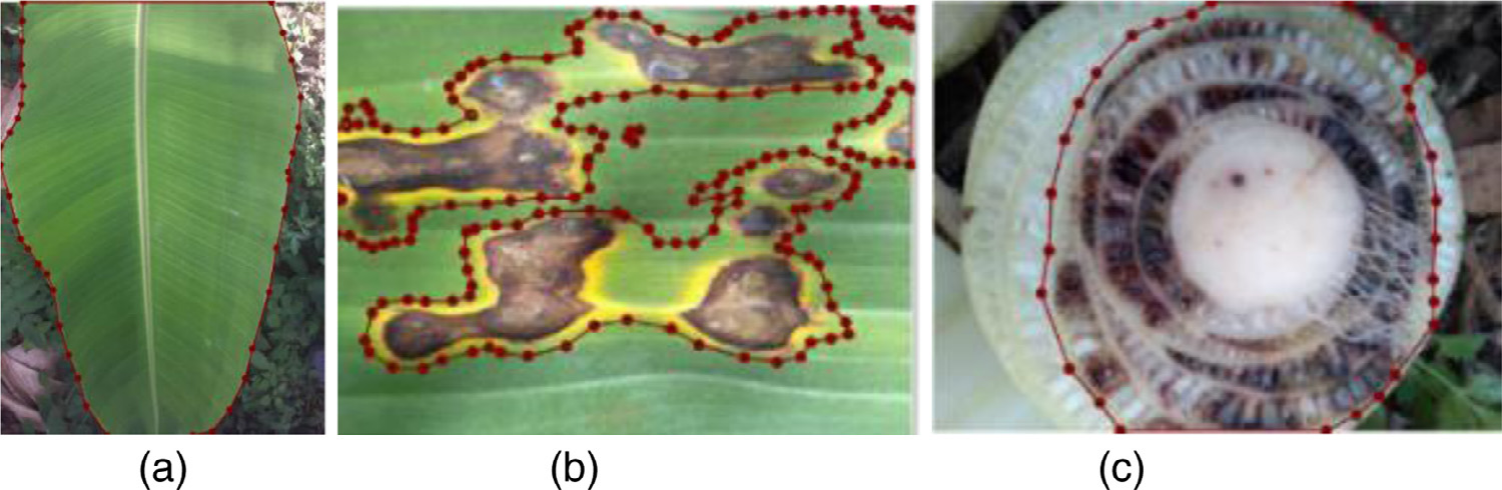
Annotated image sample of banana (a) healthy (b) Black Sigatoka (c) Fusarium Wilt Race 1- Stem.

Images were then annotated to indicate the belonging class (Healthy, Black Sigatoka, Fusarium Wilt Race 1) after the dataset had been cleaned. The clean images were given new names that included image numbers. The images were annotated for various computer vision tasks like image segmentation as shown in Fig. 2, by using the web annotation tool developed by Makerere AI Lab [4]. The curated images were then publicly shared in the Havard DataVerse repository [5]. It was vital to guarantee that the dataset did not contain images taken in extremely low lighting settings or gathered using low-quality smartphone cameras because the image quality was of the utmost importance for this dataset and would otherwise lead to bias.

## Ethics Statements

The study does not involve experiments on humans or animals.

## CRediT authorship contribution statement

**Neema Mduma:** Conceptualization, Methodology, Software, Validation, Formal analysis, Investigation, Resources, Data curation, Writing – original draft, Writing – review & editing, Visualization, Supervision, Project administration, Funding acquisition. **Judith Leo:** Conceptualization, Methodology, Writing – review & editing, Supervision, Project administration.

## Declaration of Competing Interests

The authors declare that they have no known competing financial interests or personal relationships that could have appeared to influence the work reported in this paper.

## Data Availability

Bananas Dataset Tanzania (Original data) (Dataverse).

## References

[1] Lucas S., Jomanga K. (2021). The status of banana production in Tanzania; a review of threats and opportunities. Int. J. Curr. Sci. Res. Rev..

[2] Softonic, (2023). https://visipics.en.softonic.com.

[3] Chen Q., Zobel J., Zhang X., Verspoor K. (2016). Supervised learning for detection of duplicates in genomic sequence databases. PLoS ONE.

[4] Makerere AI Lab (2023). https://github.com/AI-Lab-Makerere/web-annotation-tool.

[5] Mduma N., Leo J., Loyani L., Jomanga K., Kamara A., Msaki I., Sanga S. (2022).

